# Supplementary material for: Successful elimination of falciparum malaria following the introduction of community-based health workers in Eastern Myanmar: A retrospective analysis
Source: PLoS Med. 2023 Nov 30;20(11):e1004318. doi: 10.1371/journal.pmed.1004318 (PMC10721164; doi:10.1371/journal.pmed.1004318)
Supplement: S1 Table — (DOCX) [file pmed.1004318.s002.docx]

**S1 Table: Incidence and RDT positivity rate ratios in the rainy and cool seasons as compared to the summer**

|  | **Incidence rate ratios**  **(95% CI)** | **RDT positivity rate ratios**  **(95% CI)** |
| --- | --- | --- |
| ***P. falciparum (+mixed infections)*** |  |  |
| Summer | Reference | Reference |
| Rainy season | 2.74 (1.97 – 3.81) | 1.57 (1.17 – 2.11) |
| Winter | 1.89 (1.33 – 2.68) | 1.55 (1.13 – 2.13) |
| ***P. vivax*** |  |  |
| Summer | Reference | Reference |
| Rainy season | 1.70 (1.37 – 2.11) | 0.97 (0.79 – 1.18) |
| Winter | 1.18 (0.94 – 1.49) | 0.93 (0.75 – 1.15) |

Summer (March to April), Rainy season (May to October), Winter (November to February)
