# Supplementary material for: Successful elimination of falciparum malaria following the introduction of community-based health workers in Eastern Myanmar: A retrospective analysis
Source: PLoS Med. 2023 Nov 30;20(11):e1004318. doi: 10.1371/journal.pmed.1004318 (PMC10721164; doi:10.1371/journal.pmed.1004318)
Supplement: S2 Table — (DOCX) [file pmed.1004318.s003.docx]

**S2 Table: Active case detection**

| **Year** | **Number of villages** | **Number of tests** | ***P. falciparum (+mixed infections)*** | | ***P. vivax*** | |
| --- | --- | --- | --- | --- | --- | --- |
|  |  |  | **Cases** | **Positivity rate** | **Cases** | **Positivity rate** |
| 2011 | 22 | 4,136 | 72 | 1.74 | 39 | 0.94 |
| 2012 | 39 | 5,745 | 4 | 0.06 | 8 | 0.13 |
| 2013 | 48 | 12,781 | 11 | 0.08 | 14 | 0.10 |
| 2015 | 2 | 78 | 0 | 0 | 0 | 0 |
| 2018 | 5 | 755 | 0 | 0 | 1 | 0.13 |
| Total | 116 | 23,495 | 87 | 0.37 | 62 | 0.26 |
