# Supplementary figures and images for: Successful elimination of falciparum malaria following the introduction of community-based health workers in Eastern Myanmar: A retrospective analysis
Source: PLoS Med. 2023 Nov 30;20(11):e1004318. doi: 10.1371/journal.pmed.1004318 (PMC10721164; doi:10.1371/journal.pmed.1004318)

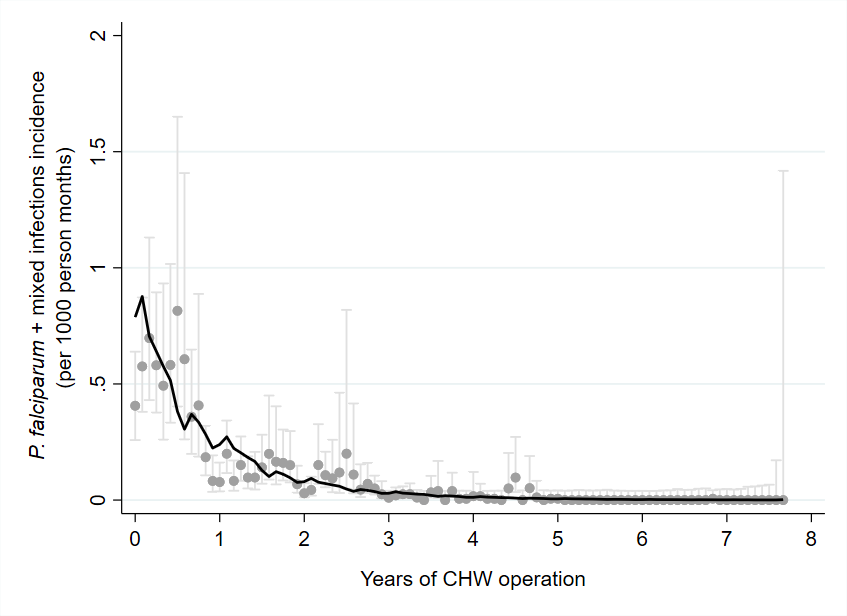

Supplement: S1 Fig — Grey dots represent observed data, the grey capped bars show the 95% CIs of each month of observed data in isolation, and the black line indicates the prediction from a mixed effects negative binomial regression model. (TIF) [file pmed.1004318.s004.tif]

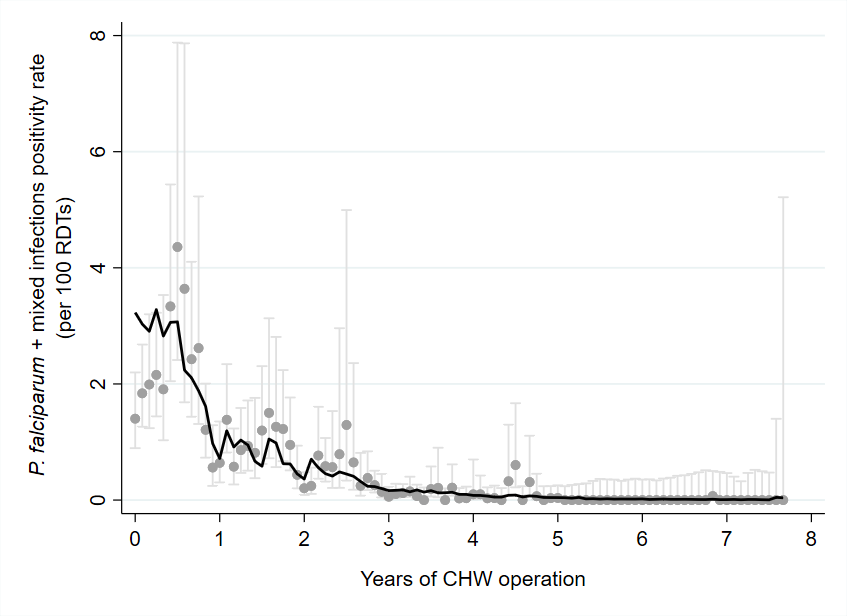

Supplement: S2 Fig — Grey dots represent observed data, the grey capped bars show the 95% CIs of each month of observed data in isolation, and the black line indicates the prediction from a mixed effects negative binomial regression model. (TIF) [file pmed.1004318.s005.tif]

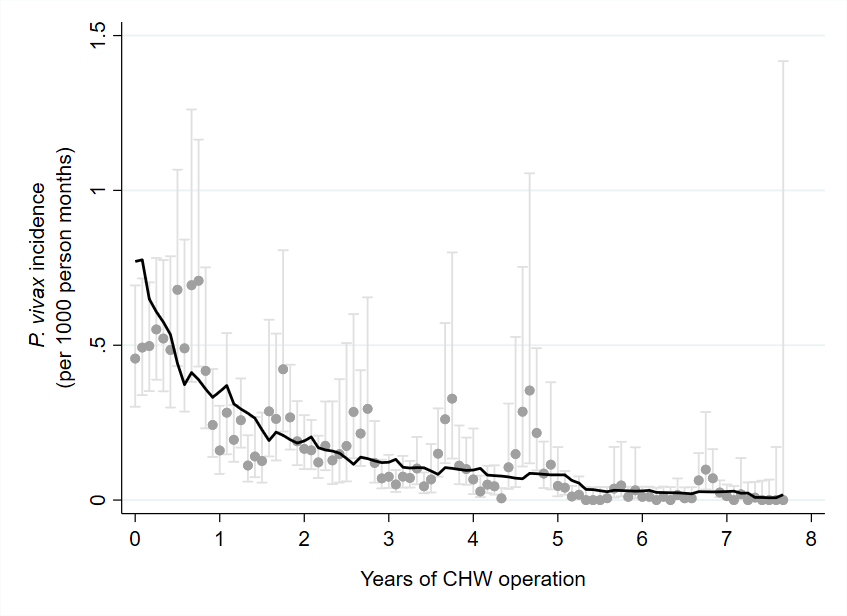

Supplement: S3 Fig — Grey dots represent observed data, the grey capped bars show the 95% CIs of each month of observed data in isolation, and the black line indicates the prediction from a mixed effects negative binomial regression model. (TIF) [file pmed.1004318.s006.tif]

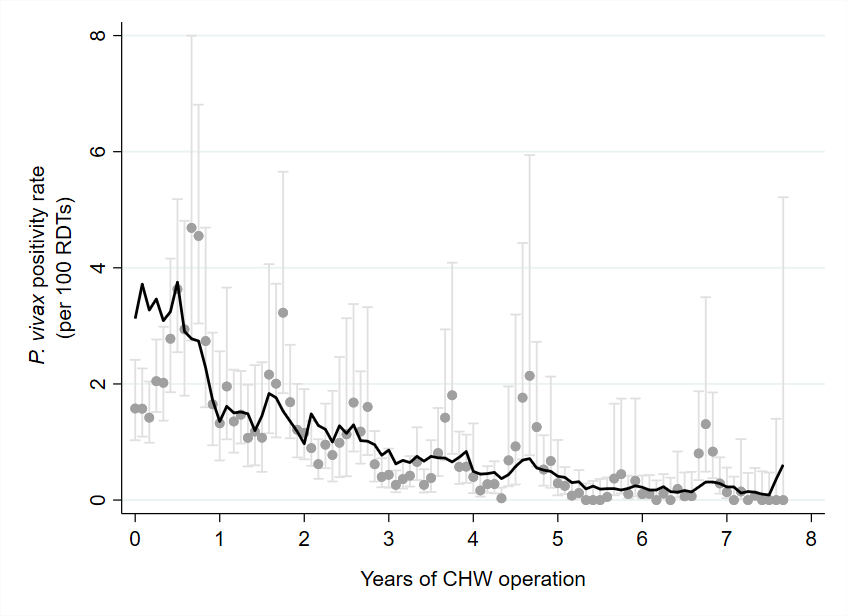

Supplement: S4 Fig — Grey dots represent observed data, the grey capped bars show the 95% CIs of each month of observed data in isolation, and the black line indicates the prediction from a mixed effects negative binomial regression model. (TIF) [file pmed.1004318.s007.tif]
