# Supplementary material for: Successful elimination of falciparum malaria following the introduction of community-based health workers in Eastern Myanmar: A retrospective analysis
Source: PLoS Med. 2023 Nov 30;20(11):e1004318. doi: 10.1371/journal.pmed.1004318 (PMC10721164; doi:10.1371/journal.pmed.1004318)
Supplement: Attachment — Submitted filename: REPLY_to_comments_from_the_academic_editor_and_reviewers.docx [file pmed.1004318.s008.docx]

**Comments from the academic editor:**

The paper presents important findings and is clearly presented and written. A key issue to address in the revision, as raised by the reviewers, is that there was no control or comparison group because this is an analysis of the implementation of a intervention program. However, there remains the question of what would we have expected to happen to malaria incidence over that period in the absence of the intervention. Other factors (e.g. climate, rainfall, other malaria intervention activities) could have contributed to the reduction over time. It seems very unlikely that factors other than the CHW intervention program would have had a major effect on malaria reduction. The authors could include some comparison data to help understand that, such as malaria trends in neighboring regions, information about rainfall or other relevant climate factors over that time, any data on vectors. This should also be addressed further in the discussion to recap for the reader why we can conclude that the malaria reduction is wholly or mostly due to the intervention.

Thank you for this important comment. We have reinforced in several places that this is an observational study and that causal inference is limited. We described the interventions as “associated with” rather than “causing” , and then argue the likely relationship. We acknowledge that unidentified confounders cannot be excluded. We acknowledge also the importance of population movement (although that would have lessened rather than augmented impact). Unfortunately the National reporting is unreliable as a comparator and unsurprisingly there are no nearby epidemiological surveys. As you rightly mentioned, “it seems very unlikely that factors other than the CHW intervention program would have had a major effect on malaria reduction”. The project period was over a relatively short period with no major climate change. We have reported rainfall in figure 1.

Other malaria intervention activities were minimal except for the distribution of LLIN. We have added a comment that indicates that LLIN may have been a contributing factor.

-----------------------------------------------------------

**Requests from the editors:**

Your manuscript has been assessed by four reviewers whose reports can be found below. As you will see from the comments, the reviewers have raised a number of concerns that need addressing. Please carefully revise the manuscript to address all comments raised.

Causal language: given the study design please use associational language in the discussion and other sections. Please check and amend throughout.

We do acknowledge this and have modified the text accordingly

The polemic about the “expensive and time-consuming activities suggested by WHO” seems inappropriate. Please rephrase or remove.

We have rephrased but it is very important to describe quite how unrealistic the current recommendations are. It is essential that donors and malaria control programmes understand the realities on the ground. Continued support for unachievable interventions diverts the limited funds away from needed activities.

For in-text reference, citations are placed within square parentheses and should precede punctuation. Please check and amend throughout. Please provide 95% CIs and p values for all results were appropriate, check and amend throughout.

For p values, please report these as p<0.001 and where higher as p=0.002 or p=0.050 (Please report all p values consistently to 3 decimal places - thousandths). Check and amend throughout.Please specify the statistical test used to determine p value.

Suggest reporting statistical information as follows for clarity for the reader “…70%; 95%CI [65%, 75%]; Suggest use of commas as opposed to hyphens (as these can be confused with negative values) to separate upper and lower bounds.

Thank you. We have addressed this.

STUDY DESIGN:

Please ensure that the study is reported according to the STROBE guideline, and include the completed STROBE checklist as Supporting Information. Please add the following statement, or similar, to the Methods: ""This study is reported as per the Strengthening the Reporting of Observational Studies in Epidemiology (STROBE) guideline (S1 Checklist).""

The STROBE guideline can be found here: http://www.equator-network.org/reporting-guidelines/strobe/

When completing the checklist, please use section and paragraph numbers, rather than page numbers."

"Did your study have a prospective protocol or analysis plan? Please state this (either way) early in the Methods section.

a) If a prospective analysis plan (from your funding proposal, IRB or other ethics committee submission, study protocol, or other planning document written before analyzing the data) was used in designing the study, please include the relevant prospectively written document with your revised manuscript as a Supporting Information file to be published alongside your study, and cite it in the Methods section. A legend for this file should be included at the end of your manuscript.

b) If no such document exists, please make sure that the Methods section transparently describes when analyses were planned, and when/why any data-driven changes to analyses took place.

c) In either case, changes in the analysis-- including those made in response to peer review comments-- should be identified as such in the Methods section of the paper, with rationale."

For all observational studies, in the manuscript text, please indicate: (1) the specific hypotheses you intended to test, (2) the analytical methods by which you planned to test them, (3) the analyses you actually performed, and (4) when reported analyses differ from those that were planned, transparent explanations for differences that affect the reliability of the study's results. If a reported analysis was performed based on an interesting but unanticipated pattern in the data, please be clear that the analysis was data-driven.

This has been addressed (Line 131-135, page 6).

TITLE:

Please revise your title according to PLOS Medicine's style. Please place the study design ("A randomized controlled trial," "A retrospective study," "A modelling study," etc.) in the subtitle (ie, after a colon).

This has been amended (Line 2, page 1).

DATA AVAILABILITY:

Please review the data availability statement.

Can you please add ‘freely ‘ to your statement “The data underlying this publication are available upon request to the Mahidol-Oxford Tropical Medicine Research Unit Data Access Committee … “

We have, but we are concerned that “freely” implies that there is no process for reviewing (and thus potentially rejecting) data requests. In fact, we have never rejected a request but it is possible. Are you sure about this word?

ABSTRACT:

Abstract Background:

The final sentence should clearly state the study aim/question.

Abstract Methods and Findings:

Please quantify the main results (with 95% CIs and p values).

Please check line 41: “.. examination rate was 1.33% (0.38–3.48%) per month.” Missing ‘95% CI’ ?

In the last sentence of the Abstract Methods and Findings section, please describe the main limitation(s) of the study's methodology.

Abstract Conclusions:

Please address the study implications without overreaching what can be concluded from the data; the phrase ""In this study, we observed ..."" may be useful.

Please interpret the study based on the results presented in the abstract, emphasizing what is new without overstating your conclusions.

Mention specific implications substantiated by the results.

Thank you: we have attended to these points (Line 31-58, page 2-3).

AUTHORS SUMMARY:

At this stage, we ask that you include a short, non-technical Author Summary of your research to make findings accessible to a wide audience that includes both scientists and non-scientists. The Avalueuthor Summary should immediately follow the Abstract in your revised manuscript. This text is subject to editorial change and should be distinct from the scientific abstract. Please see our author guidelines for more information: https://journals.plos.org/plosmedicine/s/revising-your-manuscript#loc-author-summary

Ideally each sub-heading should contain 2-3 single sentence, concise bullet points containing the most salient points from your study.

In the final bullet point of ‘What Do These Findings Mean?’ Please include the main limitations of the study in non-technical language.

We have added this (Page 4).

DISCUSSION:

Please present and organize the Discussion as follows: a short, clear summary of the article's findings; what the study adds to existing research and where and why the results may differ from previous research; strengths and limitations of the study; implications and next steps for research, clinical practice, and/or public policy; one-paragraph conclusion.

ACKNOWLEDGMENTS/ DECLARATIONS

Please remove all statements apart from acknowledgements, author contributions and abbreviations from the end of the main manuscript and include these only in the relevant parts of the manuscript submission form. Funding, competing interest, and data availability will be compiled as metadata.

REFERENCES:

Please use the "Vancouver" style for reference formatting, and see our website for other reference guidelines https://journals.plos.org/plosmedicine/s/submission-guidelines#loc-references

Please ensure that in the bibliography up to but no more than 6 author names are listed, followed by et al., in the event that more than 6 authors contribute to an individual study. Journal name abbreviations should be those found in the National Center for Biotechnology Information (NCBI) databases.

Please also ensure that any references to online-only sources include a date of accession, and in the reference list, please convert all italics to plain text.

FIGURES and TABLES

Please provide titles and legends for each individual table and figure

Figure 1: Please consider avoiding the use of red and green in order to make your figure more accessible to those with colour blindness

Table 1, 2 and 3: Please define the abbreviations.

SUPPORTING INFORMATION:

As above, please ensure that the study is reported according to the STROBE guideline, and include the completed STROBE checklist as Supporting Information. When completing the checklist, please use section and paragraph numbers, rather than page numbers. Please add the following statement, or similar, to the Methods: "This study is reported as per the Strengthening the Reporting of Observational Studies in Epidemiology (STROBE) guideline (S1 Checklist).

-----------------------------------------------------------

All these points have been attended to.

**Comments from the reviewers:**

**Reviewer #1**: This important retrospective analysis aims to assess the impact of introducing CHWs to deliver early diagnosis and treatment for malaria in hard-to-reach communities in Mon state, East Myanmar.

Comments:

"The incidence of P. falciparum malaria (including mixed infections) declined by 70% (95% CI: 65-75%) each year of CHW operation. P. vivax malaria incidence declined by 56% (95% CI: 50-62%) per year. Malaria RDT positivity rates for P falciparum and P vivax declined by 69% (95% CI: 62-75%) and 53% (95% CI: 47-59%) per year respectively."

Can the authors please attempt to assess and comment on what the malaria rates were expected to do over time without any intervention in place?

This is a good question but it is hard to answer. It is difficult to know what the malaria rates were and would be over time, because malaria data in Myanmar are notoriously unreliable, in particular in remote communities. In most remote communities there were no official health staff and fever patients were managed by unofficial health providers (locally called “quacks”) who usually did not have/use diagnostics, and treated malaria – often delayed - outside official guidelines. They did not report malaria.

As malaria transmission is low to moderate in this region, and there have been no major climatic or environmental changes, we would expect that malaria transmission would probably continue on a similar level as before without our intervention. The increasing availability of artemisinin treatment in the market, and therefore also in the hands of the quacks, might have reduced transmission somewhat over the past decades but we cannot be sure.

Only when we introduced CHWs, quality diagnosis and treatment was easy available and data were collected regularly and systematically. The uptake was high because the services were *in* the village and free of charge.

"MAM introduced CHWs in remote communities in Mon state in consultation with the Myanmar National Malaria Control Programme (NMCP)."

and

"In collaboration with the NMCP, CHWs, and MAM mobile medical teams also engaged in mass distribution of long-lasting insecticidal nets (LLINs) during the first four years of the programme. Additional LLINs were made available for newcomers to the village, migrants, and pregnant women. In addition, CHWs and MAM mobile medical teams organized health education and community engagement discussions to inform the community and seek their feedback."

In addition to the above interventions, what other factors (i.e. confounders) may be affecting the trend seen?

Besides the introduction of CHWs with RDTs, antimalarials and LLINs, there were no substantial anti-malaria interventions. The communities are very small and we would surely have noticed other substantial interventions.

It is difficult to imagine other factors that could have influenced such a large number of villages over a distance of 250 km, except for climate factors. However, the project period was short and there were no substantial climate changes.

"The introduction of CHWs providing community-based malaria diagnosis and treatment, integrated in basic health care services, in remote communities in Mon state led to a dramatic reduction in malaria."

Can the auhtors please consider whether this study design allows for causality to be inferred (i.e. 'led to')?

We agree that it does not and have replaced ‘led to’ with ‘was associated with’ (Line 56, page 2).

"RDT positivity rates and monthly blood examination rates were calculated from monthly malaria reports. Negative binomial mixed effects regression models were constructed with random intercepts and slopes to assess temporal changes in incidence and RDT positivity rates. Seasonal and geographic variations in outcomes were accounted for in the multivariate models. "

A technically appropriate and rigorous methodology has been applied.

"When modelling incidence and RDT positivity rates, the outcome was malaria (RDT-positive) results (per month, per CHW) and the primary exposure was years of CHW operation. The exposure variable for the incidence model was the population served by a CHW, and the exposure variable for the RDT positivity rate model was the number of RDTs performed."

Can the authors please clarify these models? For instance:

When modelling incidence, is it assumed that all incident cases have been identified in the population served by a CHW?

Yes-the CHW were the only source of immediate health care and the uptake of their services is high. We cannot exclude self treatment but there is no reason to believe either that this was common or that it changed during the period of observation.

When modelling RDT positivity rates, does the exposure variable assume a representative sample (i.e. such that the rate can be generalised)?

Yes – the samples were taken from the entire population and the proportions were of all cases seen by the CHWs.

The exposure variable is RDTs performed on patients who sought the care of a CHW for their fever. As mentioned above, the uptake of CHW services – and RDTs performed - was high throughout the study period. Whether the RDT positivity rates of febrile individuals who sought the care of a CHW are representative of all febrile individuals (including those who do not seek the care of a CHW) is not something we are able to test, but we think it is a reasonable assumption.

Figure 4: Can CIs for estimates please be presented on these graphs?

We have added the CI for Figure 4. However, as the figures turned out to be rather cluttered with the CI lines, we would like to suggest to keep the original graphs in the main paper and we added the figures with CI as supplementary figures.

**Reviewer #2**: Thanks for this nice documentation of malaria elimination in Mon. The story that routine diagnosis and treatment likely played a critical role in eliminating malaria there, and is likely both more effective and more sustainable than aggressive reactive approaches, is an important one. A few suggestions to strengthen it:

1) L56-58: "The World Health Organization has developed several detailed 'malaria elimination manuals' in recent years". The five references here do not actually include what I believe is the most current WHO elimination recommendations, a document called A Framework for Malaria Elimination from 2017: https://www.who.int/publications-detail-redirect/9789241511988. The framework gets rid of the ideas of discrete "phases" and instead describes elimination on a spectrum, and it rejects the idea that malaria operations should be based on the arbitrary thresholds described in this paragraph, such as shifting to different types of programs when there is 1 case per 1,000 per year - such decisions are highly context specific. The main content here - that elimination is recommended to involve targeting strong activities to foci of transmission - remains largely unchanged, but I'd suggest updating the framing.

Thank you -we have updated the text in the introduction and replaced the reference as suggested (Line 95-102, page 5).

2) L115 - PCD of course more specifically means that febrile individuals had to seek treatment from the CHW - they would not actively seek out fevers in the community. Can you confirm this is correct? Later there is a description of how the CHWs also provided ACD, but those data are presented separately?

Yes. This is correct. Febrile individuals sought diagnosis and treatment from the CHWs.

The ACD activity reported was very small scale and unlikely to have had a substantial effect on the malaria situation.

3) L126 - Is there any data on adherence to the 8 weeks of PQ?

We have no data on the adherence. However, observing the substantial decline of *P. vivax* suggests that adherence was good. We have added discussion of this important point. (Line 348-352, page 17)

4) I do not see any evaluation on reporting rates or how they varied over time - it might be reassuring to show those if available as evidence that the observed declines are reliable.

Reporting was monthly and supervised and monitored by the ‘mobile teams’ of MAM, the NGO. There was no change in this monitoring.

The very high RDT testing in 2017 and 2018 indicates that uptake of testing remained high and that the observed declines were reliable.

5) It would be interesting to know the effect of the integration of other services in 2013-2014 - e.g., did this affect testing rates?

It did, in a positive way. This was described in a cited previous publication (reference 22)

McLean ARD, Wai HP, Thu AM, Khant ZS, Indrasuta C, Ashley EA, et al. Malaria elimination in remote communities requires integration of malaria control activities into general health care: an observational study and interrupted time series analysis in Myanmar. BMC Med. 2018;16(1):183. Epub 2018/10/23. PubMed Central PMCID: PMCPMC6196466. pmid: 30343666

6) Ascribing causality is challenging because this model shows only that malaria declined over time - there is no control or pre-post comparison. If malaria were declining unrelated to CHWs (which I do not believe!), would you not find a similar result? If there are sufficient data from before substantial uptake of CHW services, I would consider trying more of an interrupted time series to show the trend in malaria in places where CHWs were not yet introduced (or where uptake was low) and then the change following introduction (or increase in uptake).

We agree and have changed the text throughout to acknowledge the limitations of our causal inference. There were no preceding data from these remote communities before CHWs were introduced, and there are not contemporaneous epidemiological data. National reporting is notoriously unreliable. We acknowledge that this is a weakness.

7) In addition, the timing of the LLIN distributions is not specified, but obviously that could be having a substantial effect in addition to the effect of treatment. Conclusions like "this tipping point was readily reached with this single intervention" don't seem supported by the fact that there were several interventions mentioned, including routine case management + ACD + LLINs. Analysis more clearly showing those latter approaches had minimal effect would be helpful.

We agree with the statement about LLIN. These only have modest benefits in this region because of early biting exophilic vectors. We have added this to the discussion with supportive references to address this point specifically (Line 334-336, page 17).

As mentioned, the ACD was too small to have had a substantial impact.

**Reviewer #3**: The MS entitled "Successful elimination of falciparum malaria following the introduction of community based health workers in Eastern Myanmar" is an excellent work that most certainly deserves and needs to be published. The conclusions reached in the MS are of utmost importance for refining public health malaria control measures and most importantly, elimination strategies in Greater Mekong Sub-region, especially in hard-to- reach and resource-poor areas, which often also are the highest malaria risk areas. The MS is well written, the data have been appropriately analysed and clearly presented.

Thank You

**Reviewer #4**: Successful elimination of falciparum malaria following the introduction of

4 community-based health workers in Eastern Myanmar

The manuscript is well written. It highlights that improved access to timely and effective treatment and vector control by community health workers can impact the malaria burden. The authors report elimination of P. falciparum malaria and a significant decline in P. vivax in the study area. The following few issues should be addressed:

1.Malaria burden data after the project is handed over to NMCP will provide information on the sustainability of the work.

Unfortunately the internal conflict in recent years has substantially disrupted NMCP and other health sector activities. And the NMCP has stopped sharing data.

2.Line 123: "To reduce transmissibility, a single dose of primaquine (0.75mg base/kg) was..." for falciparum cases. The recommended dose of primaquine as an antigametocidal is 0.25mg/kg. Why was there three times this dose used? Perhaps it is a typo error.

No, it was the National recommendation at the time-and we have now added this (we agree with the referee that 0.25mg/kg would have been better) (Line 175, page 7)

3.In the "Additional intervention" section, the mass distribution of LLINs and additional LLINs to new arrivals, migrants, and pregnant women in the first 4 years was given. This is an important measure. The author presented the number of LLINs distributed in the results. The introduction of LLINs should also be presented in the disease burden figure with the rainfall. In addition, the authors have ignored the potential impact of this intervention and should discuss it in the discussion section and conclusion.

We have added this but, as mentioned above, LLIN have only modest benefits in this region because of early biting exophilic vectors. The discussion now addresses this point specifically (Line 334-336, page 17).

Any attachments provided with reviews can be seen via the following link:

********

__________________________________________________

In compliance with data protection regulations, you may request that we remove your personal registration details at any time. (Use the following URL: https://www.editorialmanager.com/pmedicine/login.asp?a=r). Please contact the publication office if you have any questions.
